# *QuickStats*: Human Immunodeficiency Virus Disease Death Rates* Among Women Aged 45–64 Years, by Race and Age Group — National Vital Statistics System, United States, 2000–2015

**DOI:** 10.15585/mmwr.mm6637a11

**Published:** 2017-09-22

**Figure:**
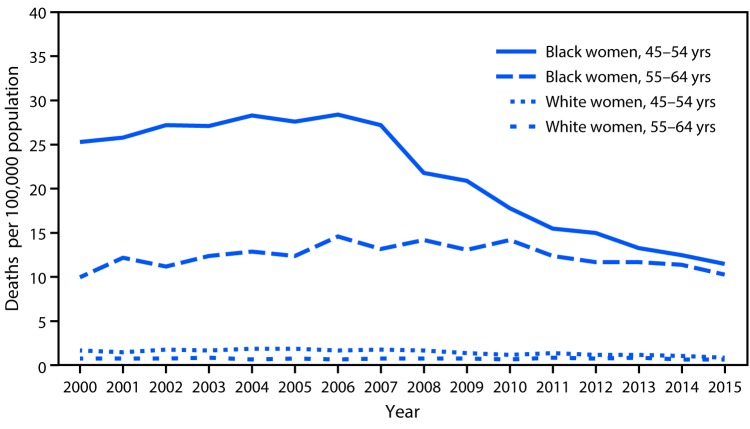
Among black women aged 45–54 years, the human immunodeficiency virus (HIV) disease death rate decreased 60% from 28.4 per 100,000 in 2006 to 11.5 in 2015. Among black women aged 55–64 years, the rate increased 42% from 10.0 in 2000 to 14.2 in 2008, before declining to 10.3 in 2015. Among white women aged 45–54 years, the rate decreased 53% from 1.9 in 2005 to 0.9 in 2015. Among white women aged 55–64 years, the rate did not change, remaining at about 0.8. Throughout the period, HIV disease death rates among black women were higher compared with rates among white women for both age groups.

**Source:** National Vital Statistics System. https://www.cdc.gov/nchs/data_access/vitalstatsonline.htm.

